# 16S rRNA Gene Amplicon Profiling of Anaerobic Bulking-Associated Prokaryotic Microbiota in a Mesophilic Expanded Granular Sludge Bed Reactor for Beverage Wastewater Treatment

**DOI:** 10.1128/MRA.00678-19

**Published:** 2019-07-25

**Authors:** Takeshi Yamada, Jun Harada, Yuki Okazaki, Tsuyoshi Yamaguchi, Atsushi Nakano

**Affiliations:** a Department of Applied Chemistry and Life Science, Toyohashi University of Technology, Toyohashi, Aichi, Japan; b Department of Civil and Environmental Engineering, National Institute of Technology, Matsue College, Matsue, Shimane, Japan; c Sumitomo Heavy Industries Environment Co., Ltd., Shinagawa, Tokyo, Japan; University of Southern California

## Abstract

Information regarding prokaryotic microbiota associated with anaerobic bulking is limited. Here, we provide 16S rRNA gene-based prokaryotic diversity profiles for anaerobic bulking and healthy granular sludge in a mesophilic expanded granular sludge bed (EGSB) reactor. These data were tabulated at the phylum level based on high-quality reads.

## ANNOUNCEMENT

Upflow anaerobic sludge blanket (UASB) and expanded granular sludge bed (EGSB) reactors are core anaerobic wastewater treatment reactors for medium- and high-strength organic wastewater (1). Healthy granular sludge formation inside these reactors is essential. However, changes in granular sludge sedimentation due to the overgrowth of specific filamentous microorganisms contribute to anaerobic bulking, leading to the outflow of granular sludge from UASB and EGSB reactors (2–4). Anaerobic bulking, identified during the startup of a full-scale mesophilic (30 to 32°C) EGSB reactor (established in Shizuoka, Japan, in February 2014) for beverage wastewater treatment, was caused by certain filamentous microorganisms. 16S rRNA gene amplicon profiles of the microbiota were generated to determine biotechnological applications for reactor recovery and anaerobic bulking prevention.

Bulking sludge and two healthy granular sludges (500 ml each) were collected from a sampling port located 2 m from the EGSB reactor bottom (reactor volume, 177 m^3^) (Table 1). The composition of wastewater supplied to the EGSB reactor, including chemical oxygen demand (COD), was measured as reported previously (3, 5, 6). Most of the COD content in the wastewater comprised carbohydrates, proteins, and some organic acids, 2,880, 457, and 922 mg COD/liter, respectively. DNA was extracted from the sludges as described previously (5). PCR amplification of the V4 region of the 16S rRNA gene was performed using Blend *Taq* polymerase (Toyobo, Osaka, Japan) and a 515F/806R primer set (7). PCR product sequences were analyzed using the MiSeq platform and MiSeq reagent kit v2 (2 × 300 bp; Illumina, San Diego, CA, USA) at the Bioengineering Lab. Co., Ltd. (Kanagawa, Japan) with six to seven technical replicates. The adaptor, index, and primer regions of each raw sequence read were trimmed using the FASTX-Toolkit v0.0.13 (8). Read sequences of ≤40 bp and with ambiguous bases and low-quality sequences (quality score, ≤Q20) were filtered out, together with their paired-end reads, using Sickle v1.33 (9). High-quality paired-end reads were merged using PEAR v0.9.10 with default settings (10). Merged sequences of ≤245 and ≥260 bp were discarded using SeqKit v0.8.0 (11). Operational taxonomic units (OTUs) of the sludge microbiota were classified using QIIME v1.9.1 (12) and the SILVA database (release 132) with 97% identity (13). The representative OTUs (>0.1%) at the phylum level within the domains *Bacteria* and *Archaea* are summarized in Table 1.

**TABLE 1:** Summary of 16S rRNA gene amplicon profiles of sludge^*a*^ microbiota

Analysis measure	Data for sample (SRA run accession no.):		
	Bulking sludge (DRR180026–DRR180032)	Healthy granular sludge A (DRR180020–DRR180025)	Healthy granular sludge B (DRR180014–DRR180019)
Sampling date	22 February 2014	27 February 2014	27 November 2015
Diversity measures			
Estimated sample coverage	0.98	0.98	0.99
No. of OTUs	427.6	363.5	420.3
Shannon diversity	4.87	4.73	5.12
Simpson diversity	0.91	0.92	0.90
Chao1 estimator	675.9	590.7	617.5
ACE^*c*^ estimator	661.2	610	626.5
No. of high-quality reads	99,979	151,163	104,000
Relative abundance (%) of bacterial and archaeal phyla			
*Euryarchaeota*	1.4	6.8	18.4
Acidobacteria	0.2	0.1	0.2
*Actinobacteria*	0.2	0.3	0.4
*Bacteroidetes*	79.7	63.8	38.4
*Chloroflexi*	4.1	15.1	10.7
*Epsilonbacteraeota*	ND^*b*^	0.1	1.6
*Firmicutes*	2.9	2.6	4.5
Nitrospirae	0.2	ND	0.1
*Proteobacteria*	7.6	8.3	21.4
*Spirochaetes*	1.8	1.4	2.6
Thermotogae	0.2	0.2	0.4
Verrucomicrobia	0.4	0.1	0.1
Others	1.3	1.1	1.3

a Bulking sludge and healthy granular sludge of a mesophilic EGSB reactor treating wastewater discharged from various lines of a beverage-producing factory.

b ND, not detected.

c ACE, abundance-based coverage estimator.

Finally, 99,979 to 151,163 high-quality reads were obtained from each sample. The bacterial and archaeal taxa were similar between bulking sludge and healthy granular sludge but differed in abundance. The predominant microorganisms (>1%) belonged to Euryarchaeota (bulking: 1.4%, healthy: 6.8 to 18.4%), Bacteroidetes (bulking: 79.7%, healthy: 38.4 to 63.8%), Chloroflexi (bulking: 4.1%, healthy: 10.7 to 15.6%), Epsilonbacteraeota (bulking: 0%, healthy: 0.1 to 1.6%), Firmicutes (bulking: 2.9%, healthy: 2.6 to 4.5%), Proteobacteria (bulking: 7.6%, healthy: 8.3 to 21.4%), and Spirochaetes (bulking: 1.8%, healthy: 1.4 to 2.6%) (Table 1). These data provide clues for addressing the issue of anaerobic bulking in EGSB reactors, e.g., by regulation of feed wastewater composition.

### Data availability.

The 16S rRNA gene amplicon data set was deposited in the DDBJ Sequence Read Archive (SRA) under accession number DRP005108 and SRA run accession numbers DRR180014 to DRR180032.
